# A genetic network mediating the control of bud break in hybrid aspen

**DOI:** 10.1038/s41467-018-06696-y

**Published:** 2018-10-09

**Authors:** Rajesh Kumar Singh, Jay P. Maurya, Abdul Azeez, Pal Miskolczi, Szymon Tylewicz, Katja Stojkovič, Nicolas Delhomme, Victor Busov, Rishikesh P. Bhalerao

**Affiliations:** 1 Umeå Plant Science Centre, Department of Forest Genetics and Plant Physiology, Swedish University of Agricultural Sciences, SE-901 87 Umeå, Sweden; 2 School of Forest Resources and Environmental Science, Michigan Technological University, Houghton, MI 49931 USA; 3 Department of Plant and Microbial Biology, University of Zürich, Zollikerstrasse 107, 8008 Zürich, Switzerland

## Abstract

In boreal and temperate ecosystems, temperature signal regulates the reactivation of growth (bud break) in perennials in the spring. Molecular basis of temperature-mediated control of bud break is poorly understood. Here we identify a genetic network mediating the control of bud break in hybrid aspen. The key components of this network are transcription factor *SHORT VEGETATIVE PHASE-LIKE (SVL)*, closely related to Arabidopsis floral repressor *SHORT VEGETATIVE PHASE*, and its downstream target *TCP18*, a tree homolog of a branching regulator in Arabidopsis. *SVL* and *TCP18* are downregulated by low temperature. Genetic evidence demonstrates their role as negative regulators of bud break. *SVL* mediates bud break by antagonistically acting on gibberellic acid (GA) and abscisic acid (ABA) pathways, which function as positive and negative regulators of bud break, respectively. Thus, our results reveal the mechanistic basis for temperature-cued seasonal control of a key phenological event in perennial plants.

## Abstract (web summary)

Molecular mechanism of how temperature-mediated control of bud break in perennial plants remains unclear. Here, the author show that transcriptional factor *SHORT VEGETATIVE PHASE-LIKE* and its downstream target *TCP18* are negative regulators of hybrid aspen bud break.

## Introduction

The survival of perennial plants in boreal and temperate ecosystems is dependent on seasonally synchronized annual growth cycles. In long-lived trees, growth ceases and dormancy is established prior to the onset of winter. Dormancy is maintained during the winter and gradual release from dormancy is followed by reactivation of growth in the spring^1^. Photoperiod and temperature are the main environmental cues regulating the seasonal synchronization of the key developmental transitions in the annual growth cycle^1^. The timing of growth cessation in the autumn, governed primarily by photoperiod signals, is mediated by the *FLOWERING LOCUS T/CONSTANS* (*FT/CO*) module in the model plant hybrid aspen^2,3^. Interaction of *FT2* with *FD-LIKE1* (*FDL1*) promotes growth under long photoperiods by ensuring high expression of the transcription factor *LIKE-AP1* (*LAP1*)^4^. *LAP1* is a positive regulator of *AINTEGUMENTA-LIKE1* (*AIL1*), a transcriptional regulator of key cell cycle genes, including D-type cyclins^5,6^. When a reduction in day length below a critical threshold permitting growth (short days/SD) is sensed, downregulation of *FT2* results in growth cessation and formation of a bud structure at the apex^2^. Buds enclose arrested leaf primordia and shoot apical meristems within protective bud scales^7^.

After growth cessation, continuation of short days induce dormancy in the buds before winter^8^. Recently, plant hormone abscisic acid (ABA) has been shown to mediate photoperiodic control of bud dormancy^7,9,10^. Once dormancy is established, buds no longer respond to growth-promotive signals^11,12^. Hence dormancy must first be released before growth can be reactivated in the buds. Dormancy release is induced by prolonged exposure to low temperature (LT) following which growth can be reactivated as visibly manifested by bud break, i.e., emergence of new leaves from the buds^11,13–16^.

The mechanisms underlying dormancy release and bud break are intimately linked but the underlying molecular mechanisms are not well understood. However, physiological and transcriptomic approaches have noted that increase in expression of gibberellic acid (GA) biosynthesis related genes and *FT2* homolog *FT1*, that are potent growth promoters and simultaneous downregulation of the components of ABA pathway coincides temporally with dormancy release and transition to bud break^17–19^. Moreover, exogenous applications of GA and ABA respectively promote and delay bud break^19,20^. However, the endogenous role of these components in bud break remains uncharacterized so far.

Earlier studies have also drawn parallels between vernalization, flowering promotion by low temperature in Arabidopsis and bud break^21,22^. For example, low temperature induces changes in the expression and chromatin status of *DORMANCY ASSOCIATED MADS-BOX* (*DAM*) genes^23,24^. Interestingly, overexpression of *DAM* genes can delay bud break^25^. While informative, these primarily based on gain-of-function approaches have not addressed the endogenous roles of *DAM* genes, and the mechanism(s) whereby they mediate control of bud break. In addition, an AP2-family transcription factor designated *EARLY BUD BREAK 1* (*EBB1*) has been identified by activation tagging in hybrid poplar, which is clearly relevant as *EBB1* overexpression and downregulation results in early and late bud break, respectively^26^. Nevertheless, whether *EBB1* acts directly in temperature control of bud break and its downstream targets remain uncharacterised. Thus, the molecular basis for translation of temperature signals into promotion of bud break remains poorly understood.

Here we report on identification of transcription factors, *SHORT VEGETATIVE PHASE-LIKE* (*SVL*) similar to Arabidopsis *SHORT VEGETATIVE PHASE* (*SVP*)^27^ a flowering time regulator and *TEOSINTE BRANCHED1, CYCLOIDEA, PCF/BRANCHED1* (*TCP18*/*BRC1*)^16^, involved in axillary bud dormancy as mediators of temperature controlled bud break. We show that *SVL* is a positive regulator of *TCP18*/*BRC1* and together they form a temperature-responsive transcriptional module that mediates control of bud break. We demonstrate that components of antagonistic ABA and GA hormonal pathways are downstream targets of *SVL* in bud break regulation. Thus, our results reveal a temperature responsive genetic network mediating bud break, a major phenological process in perennial plants.

## Results

### Transcription factor *SVL* participates in bud break control

**Fig. 1:**
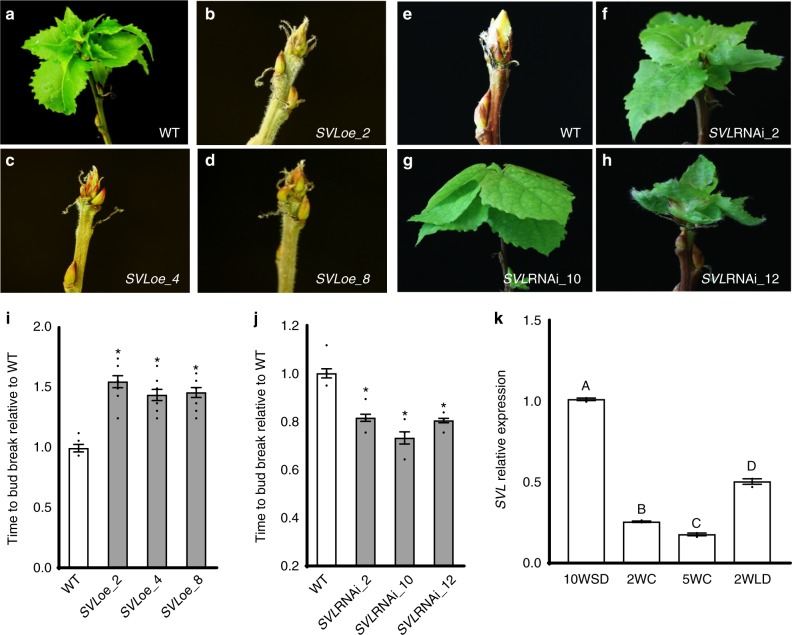
Delayed and early bud break in plants over- and under-expressing *SVL*. **a**–**d** Bud break is earlier in **a** WT plants than in three independent *SVL* overexpressing lines, designated *SVL*oe2 **b**, *SVL*oe4 **c**, and *SVL*oe8 **d**. **e**–**h** In contrast, bud break is later in WT plants **h** than in independent *SVL*RNAi lines 2 **f**, 10 **g**, and 12 **h**. **I**, **j** Time to bud break relative to wild type controls in *SVL*oe **i** and *SVL*RNAi **j** lines. Average time taken to bud break ± standard error mean (SEM), with respect to WT considered as 1, is shown with data from 10 plants from each line. Asterisks (*) indicate significant differences (*P* < 0.05) with respect to WT. **k** Low temperatures suppress *SVL* expression. Relative expression of *SVL* after 10 weeks of SD, followed by 2 and 5 weeks of low temperature (2WC, 5WC at +4 °C) and after 2 weeks subsequent exposure to long days and warmer temperatures (2WLD). *SVL* expression from three independent biological replicates ± SEM is shown relative to the reference gene UBQ with 10WSD time point set to 1. Different letters A–D over the bars indicate significant differences at *P* < 0.001. Statistical analysis was done using one way ANNOVA implying Dunnett’s/Tukey’s multiple comparison test

We screened transgenic hybrid aspen lines overexpressing transcription factors to identify genes involved in bud break. The results showed that relative to wild-type hybrid aspen plants, bud break was significantly delayed in transgenic lines overexpressing a poplar MADS box named *SVL* (*SVP-LIKE*), highly similar to *Arabidopsis* floral repressor *SVP* (Fig. 1a–d, i, Supplementary Fig. 1a). Sequence analysis indicated that SVL is similar to Arabidopsis SVP and DAM genes described in several tree species^25,28^. However, phylogenetic analysis shows that hybrid aspen SVL is more similar to SVP from *Arabidopsis* and apple (Supplementary Fig. 2) than to poplar MADS-box genes 27–29 that are homologs of *DAM* genes in peach^29^. To confirm the role of *SVL* in bud break, transgenic hybrid aspen plants with reduced *SVL* expression (*SVL*RNAi) were also generated and scored for bud break (Supplementary Fig. 1b). In contrast with *SVL* overexpressers (*SVL*oe), *SVL*RNAi lines showed early bud break compared to the control wild-type hybrid aspen plants (Fig. 1e–h, j). As *SVL*oe and *SVL*RNAi react similarly under short days, (Supplementary Fig. 3a and b), results indicate that *SVL*, has a negative role in bud break in hybrid aspen.

### LT and ABA antagonistically modulate *SVL* expression

Our data indicated that bud break was affected in *SVL* transgenics being delayed in *SVL* overexpressers and occurring earlier in *SVL* downregulated plants (Fig. 1) indicating that *SVL* mediates in bud break, a process regulated by temperature signal. Therefore, we investigated the temperature-responsiveness of *SVL* expression. *SVL* expression was significantly downregulated by exposure to low temperature and remained lower than its levels in dormant buds prior to low temperature treatment (Fig. 1k). *SVL* expression marginally increased somewhat following buds’ exposure to warm temperatures, but nevertheless remained lower than in the dormant buds. Thus, *SVL* expression is negatively regulated by low temperature. It has been reported that upon low temperature, there is an increase in the repressive marks like histone H3 lysine 27 trimethylation (H3K27me3) in the promoters of *SVL* like *DAM* genes in peach and pear^23,24^. However, we did not observe any significant increase of H3K27me3 marks at the *SVL* locus upon low temperature treatment (Supplementary Fig. 4) indicating that in contrast with other DAM genes, *SVL* suppression is not due to increase in H3K27 trimethylation at the *SVL* locus.

Like temperature signal, ABA has been implicated in bud break with exogenous application of ABA delaying bud break^20^, phenocopying *SVL* overexpresseors. Therefore we investigated whether ABA, mediates in the control of *SVL* expression. ABA application induces *SVL* expression (Supplementary Fig. 5) and moreover in the buds of transgenic hybrid aspen plants with reduced response to ABA, *SVL* expression is significantly reduced (Supplementary Fig. 6). Thus, ABA in contrast with low temperature acts positively in control of *SVL* expression.

### *FT1* and *GA20 oxidase* genes are negatively regulated by SVL

**Fig. 2:**
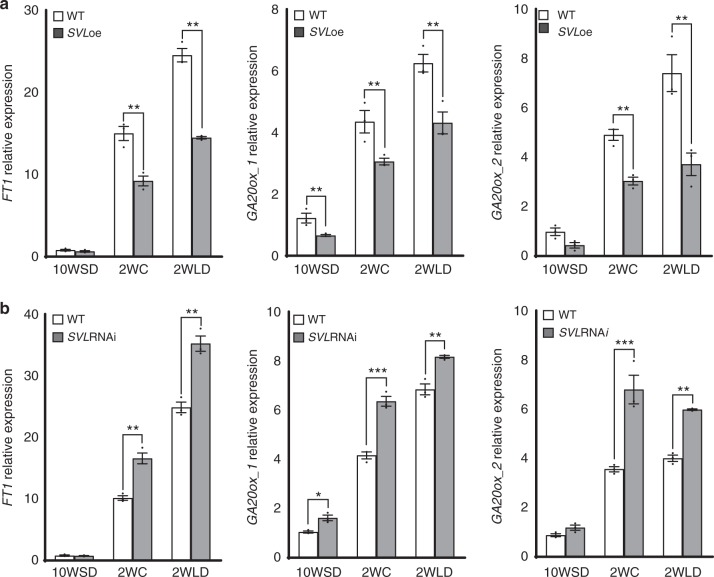
SVL negatively regulates expression of *FT1* and *GA20 oxidases* during dormancy release and bud break. Expression patterns of genes encoding *FT1*, *GA20 oxidases* (*GA20ox_1* and *GA20ox_2*) in apices of **a** WT and *SVL*oe and **b** WT and *SVL*RNAi after 10 weeks of SDs(10WSD), at the time of dormancy release (i.e. after 2weeks  of LT, 2WC), and after 2 weeks subsequent exposure to long days and warmer temperatures (2WLD). Expression values of the cited genes shown are averages for three biological replicates ± SEM, relative to the reference gene UBQ and with 10WSD time point set to 1. Asterisks indicate significant (*P < 0.05), very significant (**P < 0.005) and extremely significant (***P < 0.001) differences from corresponding controls, respectively. Statistical analysis was done using multiple *t*-tests

Low temperature enhances the expression of *FT1* and components of GA biosynthesis e.g. *GA20 oxidases* in buds mirroring the downregulation of *SVL*^6,19^. Given the growth promotive role of FT and GA’s^5,9,19^, we investigated whether *SVL* could participate in bud break by affecting *FT1* and/or GA pathway. In agreement with previous findings, low temperature-induced expression of *FT1* and *GA20 oxidases* in WT buds. In contrast, induction of *FT1* and *GA20 oxidases* was reduced in *SVL*oe buds (Fig. 2a) and, conversely, *FT1* and *GA20 oxidases* induction was enhanced in *SVL*RNAi plants relative to the wild type, after exposure to low temperature (Fig. 2b). These findings suggest a negative role for *SVL* in the induction of *FT1* and *GA20 oxidase*s expression by low temperature in hybrid aspen buds.

### SVL modulates ABA biosynthesis and signaling gene expression

**Fig. 3:**
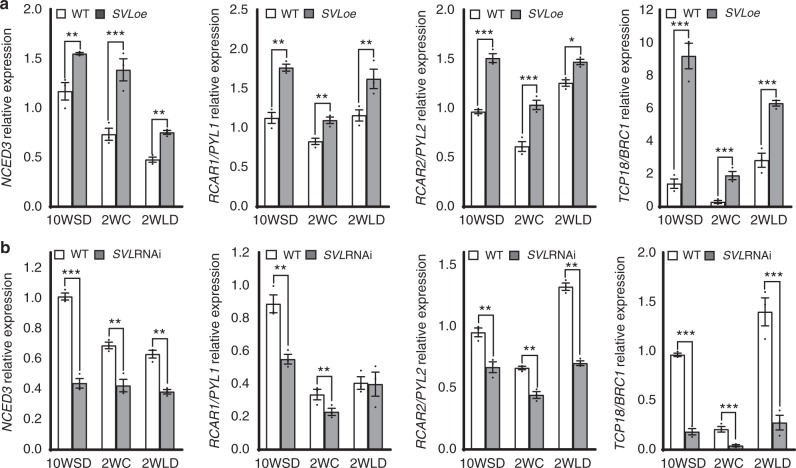
SVL positively regulates expression of the ABA biosynthesis gene *NCED3*, ABA receptors (*RCAR/PYLs*), and *TCP18*/*BRC1*-like transcription factors during dormancy release and bud break. Expression patterns of *NCED3*, *RCAR/PYL1*, *RCAR/PYL2*, and *TCP18* in apices of **a** WT and *SVL*oe and **b** WT and *SVL*RNAi after 10 weeks of SDs (10WSD), at the time of dormancy release (i.e. after 2weeks of LT, 2WC), and after 2 weeks subsequent exposure to long days and warmer temperatures (2WLD). Expression values of the cited genes shown are averages for three biological replicates ± SEM, relative to the reference gene UBQ and with 10WSD time point set to 1. Asterisks indicate significant (*P < 0.05), very significant (**P < 0.005) and extremely significant (***P < 0.001) differences from corresponding controls, respectively. Statistical analysis was done using multiple *t*-test

ABA induces *SVL* expression as shown before and exogenous application of ABA delays bud break^20^. Therefore, we investigated the transcriptional regulation of ABA biosynthesis and response machinery during bud break in the wild type and the *SVL* transgenic plants. The expression of *NCED3*, which encodes a key enzyme in ABA biosynthesis, decreased in WT buds after exposure to low temperatures. In contrast, dormant buds of *SVL*oe plants expressed *NCED3* at a higher level and did not downregulate it, relative to WT buds, in response to low temperature (Fig. 3a). Whereas in *SVL*RNAi buds, *NCED3* expression was lower and downregulated to a higher extent by low temperature than in the WT buds (Fig. 3b). Additionally, the expression of genes encoding *RCAR/PYL1* and *RCAR/PYL2*, highly similar to ABA receptors, which activate downstream signaling responses after binding ABA, is consistently higher in *SVL* overexpressers than in wild-type buds, but lower in *SVL* downregulated plants (Fig. 3a, b). Thus, ABA upregulates *SVL*, and in turn, *SVL* positively regulates ABA biosynthesis and signaling-related genes forming a feedback loop.

### SVL acts upstream of the *TCP18*/*BRC1* transcription factor

*TCP18*/*BRC1* a transcription factor that regulates axillary bud outgrowth, has been recently shown to control ABA signaling in Arabidopsis^30^. The expression of the hybrid aspen gene homologous to *TCP18*/*BRC1* is downregulated following exposure to low temperature (Supplementary Fig. 7). Therefore, we investigated whether hybrid aspen *TCP18*/*BRC1* homolog could be a target of *SVL*, by examining *TCP18*/*BRC1* expression in *SVL* transgenics. *TCP18*/*BRC1* expression was downregulated by low temperature treatment in WT buds, whereas this downregulation was attenuated in *SVL*oe buds and *TCP18*/*BRC1* expression was consistently higher in *SVL* overexpressing transgenic plants than in WT plants at all-time points (Fig. 3a). Conversely, *TCP18*/*BRC1* expression was lower in *SVL* downregulated (*SVL*RNAi) lines, suggesting that *TCP18*/*BRC1* could be a downstream target of *SVL* (Fig. 3b).

### SVL regulates *FT1*, *NCED3*, and *TCP18*/*BRC1* expression directly

**Fig. 4:**
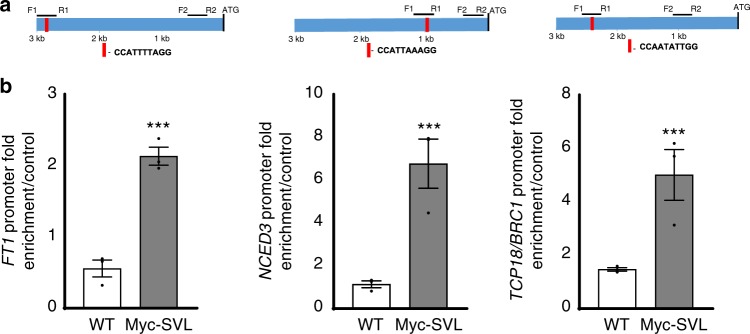
SVL binds to *FT1*, *NCED3*, and *TCP* promoters in vivo in chromatin immunoprecipitation (ChIP) assays. **a** Diagrammatic representation of *FT1*, *NCED3*, and *TCP18*/*BRC1* promoters showing the CArG motif and their positions within a 3 kb region. F1–R1 indicates positions of DNA fragments used to assess DNA–protein interactions in ChIP assays, and F2–R2 indicates positions of DNA fragments with no CArG motif used as negative controls in the assays. **b** Enrichment of the DNA fragments containing the CArG motif quantified by ChIP-q-PCR. Presented values were first normalized by their respective input values, then fold enrichments in WT and Myc-SVL plants relative to negative controls were calculated. Bars show an average values from three independent biological replicates ± SEM. Asterisks indicate significant (*P < 0.05), very significant (**P < 0.005) and extremely significant (***P < 0.001) differences from corresponding controls, respectively. Statistical analysis was done using *t*-test

The presented results showed that *SVL* mediates in temperature control of bud break and expression of growth promoters and repressors, including *FT1*, *GA20 oxidases*, *NCED3, RCAR/PYL*, and *TCP18*/*BRC1*. As *SVL* is a transcriptional regulator, we investigated which of these genes are direct downstream targets of *SVL* by chromatin immunoprecipitation (ChIP)-RT–PCR experiments on DNA isolated from shoot apices of transgenic hybrid aspen plants expressing Myc-tagged SVL (Myc-SVL) and WT control. We found clear evidence for binding of SVL in the promoters of *FT1*, *NCED3*, and *TCP18*/*BRC1* (Fig. 4) all of which contain a CArG motif known to be a target site for MADS-box transcription factors. In contrast, no evidence of SVL binding to promoters of *GA20 oxidase* (1 and 2) or *RCAR/PYL* genes was detected indicating that SVL indirectly affects the expression of these genes (Supplementary Fig. 8).

### Reduction of GA represses early bud break in *SVL*RNAi lines

**Fig. 5:**
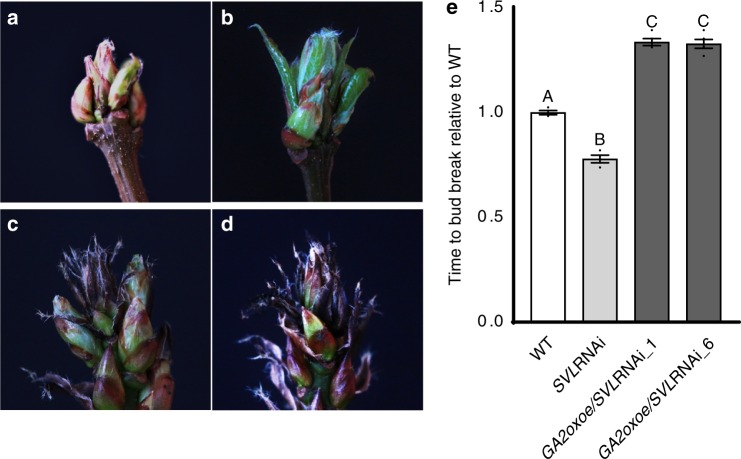
Overexpression of *GA2 oxidase* represses early bud break in *SVL*RNAi lines. **a**–**d** Bud break is earlier in **a** WT and **b** *SVL*RNAi plants than in **c**, **d** two independent lines overexpressing *GA2 oxidase* in a *SVL*RNAi background, designated *GA2oxoe*/*SVL*RNAi_1 and 6, respectively. **e** Time to bud break relative to WT controls for *SVL*RNAi plants, and lines overexpressing *GA2 oxidase* in a *SVL*RNAi background. Average time taken to bud break ± SEM, with respect to WT considered as 1, is shown with data from 10 plants from each line. Different letters A–C over the bars indicate significant differences at *P* < 0.001. Statistical analysis was done using one way ANNOVA implying Tukey’s multiple comparison test

Early bud break in *SVL*RNAi plants is correlated with enhanced expression of GA biosynthesis in these plants relative to wild type suggesting that the GA pathway could be a downstream target of *SVL* in bud break regulation. We tested this hypothesis by generating *SVL*RNAi plants overexpressing a poplar *GA2 oxidase* (Supplementary Fig. 9) with known ability to reduce GA levels^31^. We then compared the bud break of *SVL*RNAi with *SVL*RNAi expressing *GA2 oxidase* (Fig. 5). Our data show that *GA2 oxidase* expression repressed the early bud break phenotype of *SVL*RNAi transgenic lines. These results along with gene expression data strongly support that GA pathway is a downstream target of *SVL* in temperature controlled bud break.

### Overexpression of *RCAR/PYL1* and *TCP18*/*BRC1* delays bud break

**Fig. 6:**
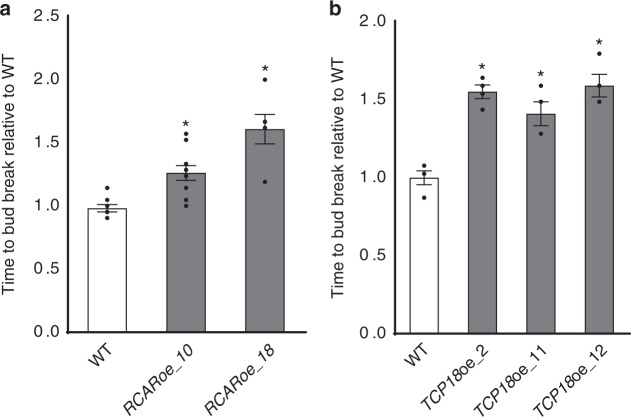
*RCAR/PYL1* or *TCP18*/*BRC1* overexpression delays bud break in hybrid aspen. Time to bud break relative to WT controls for **a** *RCAR/PYL1*oe and *TCP18*/*BRC1*oe **b** plants. Average time taken to bud break ± SEM, with respect to WT considered as 1, is shown with data from 10 plants from each line. Asterisks (*) indicate significant differences (*P* < 0.01), with respect to WT. Statistical analysis was done using *t*-test

The expression of hybrid aspen *TCP18*/*BRC1* and *RCAR/PYL* ABA receptors is affected in *SVL* transgenics and like *SVL*, *TCP18/BRC1* and *RCAR/PYLs* are downregulated in the buds after exposure to low temperature. Therefore we hypothesized that bud break involves the downregulation of *TCP18/BRC1* and *RCAR/PYLs*. We tested this hypothesis by generating transgenic plants that would maintain high levels of *RCAR/PYL1* and *TCP18*/*BRC1* then investigated bud break in these genotypes (Supplementary Fig. 10). In both, *RCAR/PYL1* and *TCP18*/*BRC1* overexpressers, bud break was significantly delayed compared to wild type control plants (Fig. 6) indicating that *RCAR/PYL1* and *TCP18*/*BRC1* have repressive roles in bud break regulation.

## Discussion

The timing of bud break in spring is critical for the survival of perennial trees growing in temperate and boreal ecosystems as premature bud break can lead to fatalities from cold snaps occurring early in the spring. Conversely, later than optimal bud break reduces the competitiveness of these trees. Here we present molecular framework underlying the regulation of bud break by temperature signal.

By screening for mediators of temperature regulation of bud break, we identified *SVL*, a MADS box transcription factor. Although *SVL* shows similarity to previously described *DAM* genes, it clusters closer to *SVP* in Arabidopsis and apple than to hybrid aspen or peach *DAM* genes. Nevertheless, high degree of similarity between SVP, SVL, and DAM suggest that they comprise a larger sub-family of MADS box genes. Our results indicate that *SVL* expression is downregulated in hybrid aspen buds after low temperature treatment. Application of both gain- and loss-of-function approaches confirmed *SVL*’s role as an endogenous mediator of temperature signals and its function as a negative regulator of bud break. SVL is a member of MADS box family of transcription factors that often form homo and heteromeric complexes. Loss-of-individual MADS box proteins results in perturbation of these complexes leading to various phenotypes^32^ and this maybe the case in *SVL* downregulated hybrid aspen plants as well. Downregulation of *SVL* described here is similar to the downregulation of the floral repressor *FLC*, also a MADS-box protein, in Arabidopsis^33,34^ during vernalization (promotion of flowering by prolonged exposure to low temperature) and those of other *DAM* genes associated with bud break^35–37^. In vernalization, *FLC* is silenced by increases in histone H3 lysine trimethylation, resulting in flowering^38^. Similarly, exposure to low temperature results in increases in H3k27 trimethylation of *DAM* loci that have been implicated in bud break in some plants^23^, highlighting the resemblance between bud break and vernalization. However, we have not detected any significant change in H3K27me3 marks at the *SVL* locus of hybrid aspen following low temperature treatment (Supplementary Fig. 4). Instead, downregulation of ABA pathway, a positive regulator of *SVL*, upon exposure to extended low temperature, could underlie downregulation of *SVL* expression. Thus, the expression of *SVL* during bud break is distinct from that shown for *FLC* in Arabidopsis during vernalization or other *DAM* like genes during bud break.

*SVL* regulates hormonal pathways that act antagonistically in bud break. *SVL* directly and indirectly promotes expression of genes encoding *NCED3* (a key ABA biosynthesis enzyme) and *RCAR/PYL* ABA receptors, respectively. Both of these genes are downregulated in response to low temperature, like *SVL*, and their response to low temperature is modulated in *SVL* transgenics. Interestingly, while *SVL* positively regulates ABA pathway, ABA itself promotes *SVL* expression. Thus ABA and *SVL* form a re-enforcing loop that acts to delay bud break. In contrast with its positive effects on ABA synthesis and signaling-related genes, we obtained clear indications that *SVL* represses GA pathway. *Inter alia*, low temperature induces the expression of *GA20 oxidase*, a key GA biosynthesis gene. The low temperature effect on *GA20 oxidase* expression is modulated by *SVL* since *SVL* overexpression and silencing respectively weakened and strengthened its induction in response to low temperature. These observations suggested that control of bud break is mediated by *SVL* acting antagonistically on ABA and GA pathways. This hypothesis was supported by the subsequent genetic analysis of bud break in plants in which ABA or GA pathway were modulated. Bud break was delayed in plants overexpressing *RCAR/PYL* and enhancing *GA2 oxidase* (which catalyzes degradation of GA) expression suppresses the early bud break phenotype of *SVL*RNAi plants. Taken together, these results explain why *SVL* overexpression delays bud break and its downregulation has the opposite effect of promoting early bud break. Thus, extended low temperature promotes bud break by downregulating *SVL* expression thereby relieving the repressive effect of ABA and promoting the GA pathway’s positive effect downstream.

In Arabidopsis and other plants e.g. pea, signals or events e.g. decapitation, that activate axillary bud outgrowth also induce downregulation of *TCP18*/*BRC1*^16,39^. Moreover, *TCP18/BRC1* transcription factor has been demonstrated to act as a negative regulator of axillary bud outgrowth by controlling bud activation potential^16,40,41^. Transcriptional analysis indicated that hybrid aspen homolog of Arabidopsis *TCP18/BRC1* was downregulated upon exposure to low temperature, like *SVL*. Moreover, our data indicated that *TCP18*/*BRC1* was a direct target of SVL and its expression was altered in *SVL* transgenics indicating a role as a negative regulator of bud break, a hypothesis supported by analysis of *TCP18*/*BRC1* transgenics. Although in *Arabidopsis*, *SVP* has not been implicated in bud dormancy or in regulation of *TCP18/BRC1,* our data now reveals a role for  *TCP18*/*BRC1* in SVL mediated control of seasonal growth in tree.

In Arabidopsis, *TCP18/BRC1* has an additional role in repressing *FT*-mediated promotion of flowering in axillary buds by binding *FT*^40^. It is noteworthy that the expression of *FT1* in hybrid aspen buds is induced simultaneously with the downregulation of *SVL* and *TCP18*/*BRC1* by low temperature. As *FT1* can act as a positive regulator of seasonal growth^3,5^, we propose that suppression of *TCP18*/*BRC1* by low temperature would serve to prevent *TCP18/BRC1* from antagonizing *FT*-mediated promotion of bud break. *SVL* can directly bind to the *FT1* and *TCP18*/*BRC1* promoters, and *SVL* has opposite effects on *FT1* and *TCP18*/*BRC1* expression (as it does on GA and ABA pathways). *SVL* regulation of *FT1* described here has similarities with the proposed regulation of *FT* homolog by *SVL* related *DAM* genes in leafy spurge indicating the conservation of this mechanism^42^. Thus, by downregulating *SVL*, low temperature would concomitantly induce *FT1* expression and downregulate *TCP18*/*BRC1*, resulting in a positive feedforward loop that enhances the potential for bud break.

**Fig. 7:**
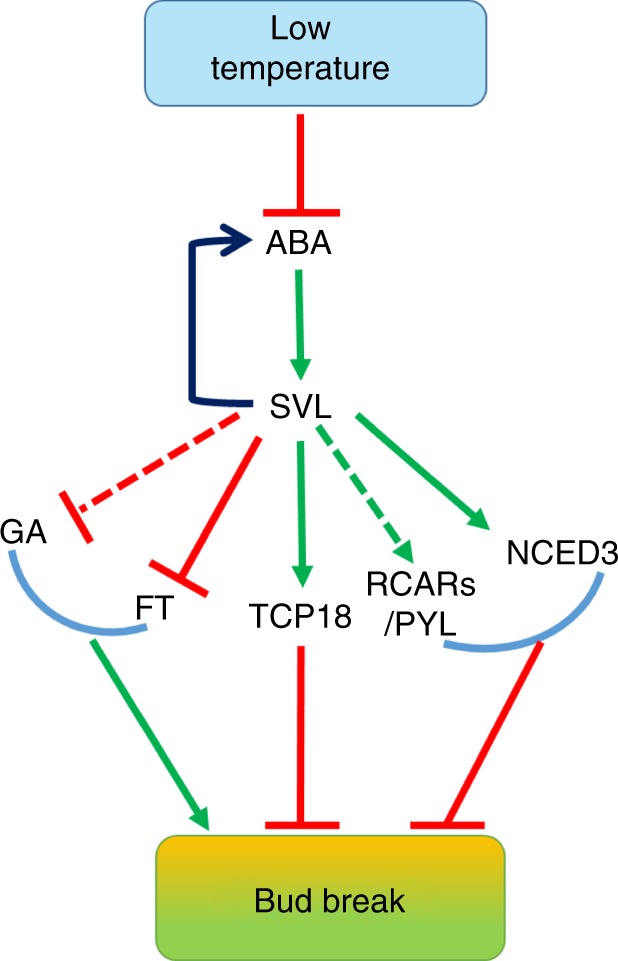
Hypothetical model integrating components involved in bud break. Low temperature reduces ABA levels and suppresses *SVL* expression, leading to induction of *FT1* expression and GA biosynthesis, which promotes bud break. In the absence of low temperatures, high levels of *SVL* expression induce *NCED3* and *RCAR/PYL*, thereby maintaining high ABA levels and sensitivity in buds, ensuring that they remain dormant. *SVL* subsequently induces *TCP18*/*BRC1*, suppressing bud break. Low temperatures trigger reduction in *SVL* expression and its suppressive effects, followed by bud break

In contrast with photoperiodically controlled growth cessation, temperature signals control bud break. Although *DAM* genes^25,36^ and *EBB1*^26^ have been implicated in this process, their roles and modes of action in bud break are not entirely clear and a molecular framework underlying bud break has not emerged so far. We identified transcription factor *SVL* and its several targets: *TCP18* and components of the antagonistically acting ABA and GA signaling pathways and elucidated their role in bud break. We propose that *SVL* and its downstream targets form a genetic network underlying the temperature-mediated control of bud break in hybrid aspen as summarized in the model (Fig. 7). According to this model, the extended cold temperature signal down regulates *SVL* and its targets (e.g. *TCP18*/*BRC1* and *RCAR/PYL)* together with simultaneous upregulation of *FT1* and the GA pathway could enhance the potential for bud break.

The antagonistic roles of ABA and GA in bud break identified here are reminiscent of their similar antagonistic actions in control of seed germination; a process inhibited by ABA and promoted by GA^43^. Moreover, low temperature treatment promotes germination of seeds as well as bud break^12^. Thus, antagonistic action between ABA and GA has been harnessed through evolution as a regulatory module in the control of dormancy and post-dormancy processes mediated by temperature signals in both seeds and buds that are crucial lifecycle adaptations to seasonal climatic changes.

The Arabidopsis homologs of *SVL* and *TCP18*/*BRC1* transcription factors, *SVP* and *TCP18*/*BRC1*, respectively, are involved in floral transition and axillary bud outgrowth^16,40,44^. Floral transition is morphogenetically distinct from phenological traits, such as apical bud dormancy and bud break. However, there are commonalities in environmental cues that regulate these developmental events which may explain why similar genetic pathways appear to have been harnessed in the adaptive regulatory machinery involved^35,45^. Our results provide an example of the utilization of flowering regulators in phenological events by external cues. We have previously shown that tree orthologs of *APETALA1* (*AP1*) are mediators of photoperiodic control of seasonal growth^5^ and show here that *SVL*-*TCP18*/*BRC1* plays a similar role in temperature control of bud break. Notably, in addition to vegetative cycles, trees undergo floral transition and floral buds are also subject to dormancy and bud break. Thus, the use of signaling components homologous to regulators of floral transition in bud break control may allow perennials to integrate the two processes, flowering and bud break, in floral buds (when trees eventually acquire flowering competence at maturity) by using common signaling components, a possibility that warrants further analysis.

## Methods

### Plant material and growth conditions

Hybrid aspen (*Populus tremula x tremuloides*) clone T89 (wild type/WT) and the transgenic plants described below were cultivated in half-strength MS medium (Duchefa) under sterile conditions for 5 weeks then transferred to soil and grown for another 4 weeks in the greenhouse (16 h photoperiods, 22 °C and 66% relative humidity). Further plants were grown in growth chambers and initially grown under long day (LD) conditions (16 h, 20 °C light/8 h, 15 °C cycles) for 1 week for acclimatization and subsequently under short day (SD) conditions (8 h, 20 °C light/16 h, 15 °C dark cycles) for 10 weeks. Responses to SDs were determined by monitoring bud set and plant growth. After 10 weeks of SDs, plants were exposed to low temperatures (4 °C) for 5 weeks to break dormancy and subsequently to the warm LD conditions (LD/WT). Bud break was scored when bud swelling and emergence of green leaves were observed. Apex samples were taken for expression analysis: after plants had ceased growth and developed dormancy, i.e. after 10 weeks of short days (10WSD) and after; both 2 weeks (2WC) and 5 weeks (5WC) of exposure to low temperature (4 °C) to induce dormancy release; and followed by two weeks after the transfer to long day/warm temperature conditions (2WLD). Pictures of apices were taken using a Canon EOS digital camera to monitor bud burst.

### Generation of plasmid constructs

Full-length *Myc-SVL* (containing 3X Myc sequence), *RCAR/PYL*, *TCP18/BRC1*, and *GA2-oxidase* cDNAs were amplified using cDNA prepared from mRNA extracted from hybrid aspen apices as templates and primers listed in Supplementary Table 1. *SVL*, *RCAR/PYL*, and *TCP18*/*BRC1* cDNAs were cloned into the pENTR/D-TOPO donor vector (Invitrogen) and transferred into the pK2GW7/pH2GW7 plant transformation vectors^46^, which contains a CaMV3S promoter to generate plasmids designated pK2GW7-Myc-*SVL*, pK2GW7-*RCAR*, pK2GW7-*TCP18*, and pH2GW7-*GA2 oxidase*, respectively, which were subsequently transformed into Agrobacterium strain GV3101pmp90RK^47^. To generate a *SVL*-RNAi construct, a 156 bp fragment was amplified using primers listed in Supplementary table and full-length *SVL* cDNA as template. The amplified fragment was cloned into pENTR/D-TOPO then transferred into the plant transformation vector pK7GWIWG2 (I) containing a CaMV3S promoter to generate a *SVL*-pK7GWIWG2 (I) construct. ABA insensitive transgenic plants (*abi1-1)* developed earlier^48^ were used in the study.

### Phylogenetic analysis

Protein sequences collected by the best BLAST match for SVP from the Popgenie database (http://popgenie.org) or NCBI were aligned and a phylogenetic tree developed using MEGA7^49^. The evolutionary history was inferred using the neighbor-joining method with 1000 bootstrap replicates. The evolutionary distances were computed using the Poisson correction method with units of the number of amino acid substitutions per site.

### Plant transformation and screening of transgenic lines

Hybrid aspen was transformed with the vectors pK2GW7- Myc-*SVL*, *SVL*-pK7GWIWG2, pK2GW7-*RCAR*, and pK2GW7-*TCP18* via *Agrobacterium*-mediated transformation^5^ to generate transgenic lines. *SVL*RNAi lines were used as background to transform pH2GW7-*GA2 oxidase*. For screening of transgenic lines leaf samples were taken from each line and used for Protein/RNA isolation, which were further used for western blotting and q-PCR analysis. To check expression of off targets in SVL downregulated lines (*SVL*RNAi) expression of selected genes was checked by q-PCR (Supplementary Fig. 1d). Putative off target genes were selected on basis of protein/nucleotide homology.

### ABA treatment of buds

For analysis of ABA response, apices were cut and placed in MS medium with or without 50 mM ABA and were sampled and used for analysis^9^.

### RNA isolation and quantitative real-time PCR analysis

Total RNA was extracted from samples of tissues, all taken at 2 pm, using a Sigma Spectrum Total Plant RNA isolation kit. Portions (10 µg) of total RNA were treated with RNase-Free DNase (Qiagen) and cleaned using an RNeasy® Mini Kit (Qiagen). One micrograms of the RNA from each sample was used to generate cDNA using an iScript cDNA synthesis kit (BioRad). Selected (UBQ-like) reference genes were validated using GeNorm Software. qRT–PCR analyses were carried out with a Roche LightCycler 480 II instrument and relative expression values were calculated using the ∆Ct method^6^. A complete list of primers used in the RT–PCR analysis is presented in Supplementary Table 1.

### Chromatin immunoprecipitation assays

The chromatin immunoprecipitation (ChIP) assays were carried out as previously described with some modifications^50,51^. Briefly, apices from actively growing hybrid aspen plants were collected and immersed in cross-linking buffer containing 1% formaldehyde and kept under vacuum for 20 min, and then glycine was added to a final concentration of 0.125 M to stop the cross-linking. Cross-linked samples were rinsed 3–4 times with water, frozen in liquid nitrogen and stored at −80 °C. Tissue samples (c.a. 1 g) were ground into fine powder and suspended in precooled nuclei isolation buffer, gently vortex-mixed to visual homogeneity then filtered through two layers of Miracloth. The homogenized, filtered mixtures were then centrifuged and the pellets obtained were re-suspended in nuclei lysis buffer. Chromatin was sheared to about 0.3–0.5 kb fragments by sonication (Bioruptor UCD-300, Diagenode). After sonication, the samples were centrifuged again to remove cell debris and each supernatant was transferred to a new tube (after retaining 10% of each sonicated sample used as Input DNA control in the Q-PCR analyses. After centrifugation, the supernatant was precleared with 40 µl protein A-magnetic beads for 60 min at 4 °C with gentle agitation and shaking. Fifteen micrograms of anti-MYC monoclonal antibody (Abcam, Cambridge, UK, Cat no. ab32; GR255064) was added to each supernatant and the resulting mixtures were further incubated overnight at 4 °C. Protein A-magnetic beads (Dynabeads, Invitrogen) were then added again and incubation was continued for 2 h. The magnetic beads were washed two times each with low salt buffer, high salt buffer, LiCl buffer, and TE buffer. The immunocomplexes were collected from beads with 250 µl of elution buffer and incubated at 65 °C for 20 min with agitation. 0.3 M NaCl was added to each tube (and Input DNA control) and cross-linking was reversed by incubation at 65 °C overnight. Residual protein was degraded by incubation with 20 mg of Proteinase K in 10 mM EDTA and 40 mM Tris-HCl, pH 8.0, at 45 °C for 1 h. After proteinase treatment precipitated DNA was purified using a ChIP DNA clean and concentrator kit according to the manufacturer’s protocol (Zymo Research Corp.). Both immunoprecipitated and input DNA were analyzed by real-time PCR using a Light Cycler instrument (Roche Applied Science). All buffers used were prepared following previous report^50^.

### ChIP-seq experiment

For the ChIP-seq experiment the apical buds of three biological replicates were collected from WT plants after 10 weeks in SD (10WSD) and after an additional 4 week cold (4WC) treatment. ChIP assays were carried described above. Anti-Trimethyl-Histone H3 (LysK27) (Millipore, Cat No. #07-449) and Anti-Histone H3 (Abcam, Cat No. ab1791) antibodies were used for chromatin immunoprecipitation. Ovation Ultralow IL Multiplex System I (Part No. 0304, NuGEN) was used to generate the sequencing libraries according to the product instructions. Pair end sequencing was done by BGI-Tech. Sequencing reads were processed following the guidelines described at http://www.epigenesys.eu/en/protocols/bio-informatics/1283-guidelines-for-rna-seq-data-analysis. Briefly, reads quality was first assessed using FastQC (http://www.bioinformatics.babraham.ac.uk/projects/fastqc/), v0.11.4. Reads mapping to ribosomal RNA (rRNA) were quantified and filtered using SortMeRNA^52^ (v2.1;settings --log --paired_in --fastx--sam --num_alignments 1) using the rRNA sequences provided with SortMeRNA. Reads were then filtered to remove adapters and trimmed for quality using Trimmomatic^53^ (v0.36; settings TruSeq3-PE-2.fa:2:30:10 SLIDINGWINDOW:5:20 MINLEN:50). After every filtering step, FastQC was run again to ensure that no technical artefacts were introduced. Reads were then mapped to the hybrid aspen genome (*Populus tremula* *×* *tremuloides*, clone T89) using STAR^54^ with settings --outQSconversionAdd −31 --outReadsUnmapped Fastx. Reads were later on remapped using BWA-MEM^55^ with default settings to comparable results. Peaks were called genome wide using MACS2^56^ with the following non-default parameters: -f BAM -g 2.7e8 -s 45 --verbose 3 --nomodel --shiftsize 100 --to-large --keep-dup all, on sequencing libraries down-sampled to 10 million PE reads. This down-sampled library depth (10 M) had been estimated by an ad hoc saturation/rarefaction analysis based on the number of peaks identified by MASC2 in varied subsets of the original dataset. These downstream analyses (peak-calling, saturation, etc.) were solely used to estimate the fraction of the genome mapped under the different growing conditions. The obtained ratios were used as part of the data normalization for the analysis of the SVL locus histone methylation status.

Reads mapped to the sequence of SVL gene including 1 kb upstream and downstream region were extracted from the alignment. Coverage in the above region was calculated, log2 transformed and corrected for the abundance differences between samples (i.e. the fraction of the genome mapped under the different growing conditions in the 10 M PE read subset; the latter selection addressing any library size factor scaling otherwise required). Finally, the H3K27me3 abundance was normalized by H3 abundance. These were used to compare differences in histone methylation between the two time points, using R^57^ and Bioconductor^58^.

### Code availability

Details about the software used in the ChIP-Seq analysis including the parameters used can be found in the description of the methods above. The R script to reproduce the SVL gene locus analysis is available from UPSCb GitHub repository https://github.com/UPSCb/UPSCb/tree/master/manuscripts/Singh2018.

## Electronic supplementary material

Supplementary Information

## Data Availability

The authors declare that all data supporting the findings of this study are included in the main manuscript file or Supplementary Information or are available from the corresponding author upon request. The data necessary to reproduce the SVL gene locus analysis is available from UPSCb GitHub repository (https://github.com/UPSCb/UPSCb/tree/master/manuscripts/Singh2018) in the form of bam files. The bam files are subset to the SVL +1 kb upstream/downstream locus only.
