# Supplementary material for: A genetic network mediating the control of bud break in hybrid aspen
Source: Nat Commun. 2018 Oct 9;9:4173. doi: 10.1038/s41467-018-06696-y (PMC6177393; doi:10.1038/s41467-018-06696-y)
Supplement: Supplementary file 1 — Supplementary Information [file 41467_2018_6696_MOESM1_ESM.pdf]

# A genetic network mediating the control of bud break in hybrid aspen

Singh *et al.*

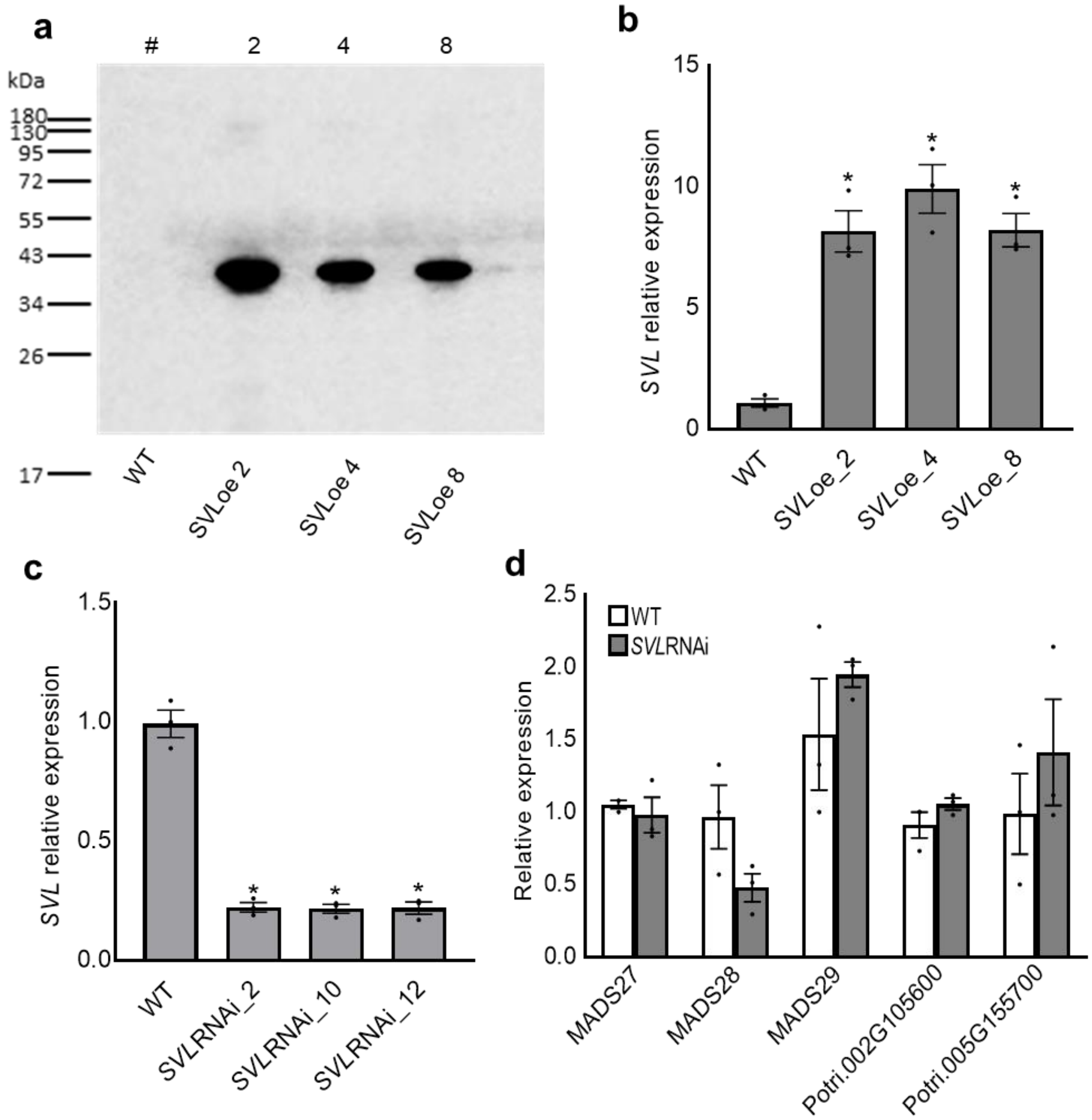


**Supplementary Figure 1.** ***SVL* expression in *SVL* overexpressing and RNAi plants. a** Western blot showing expression of Myc-SVL in WT and *SVL* overexpressing transgenic lines (2, 4 and 8). C-Myc antibody was used to probe the blots. Molecular mass markers (kDa) shown on the left **b** *SVL* expression in WT and three independent *SVL*oe lines (2, 4 and 8). **c** *SVL* expression in WT and three independent *SVL*RNAi lines (2, 10 and 12). RT-PCR analysis was performed to analyze *SVL* expression in WT and *SVL* transgenic plants. Expression of *SVL* is plotted relative to the reference gene UBQ and normalized to transcript levels in WT controls (average for three biological replicates ± SEM). Asterisks (*) indicate

significant difference at, P<0.001 compared to WT, calculated using t-test. **d** Relative expression of possible off targets of *SVL* downregulation by RNAi. Off targets (*MADS27*, *MADS28*, *MADS29*, *Potri.002G105600* and *Potri.005G155700*) were selected on basis of closest protein/nucleotide homology to SVL.

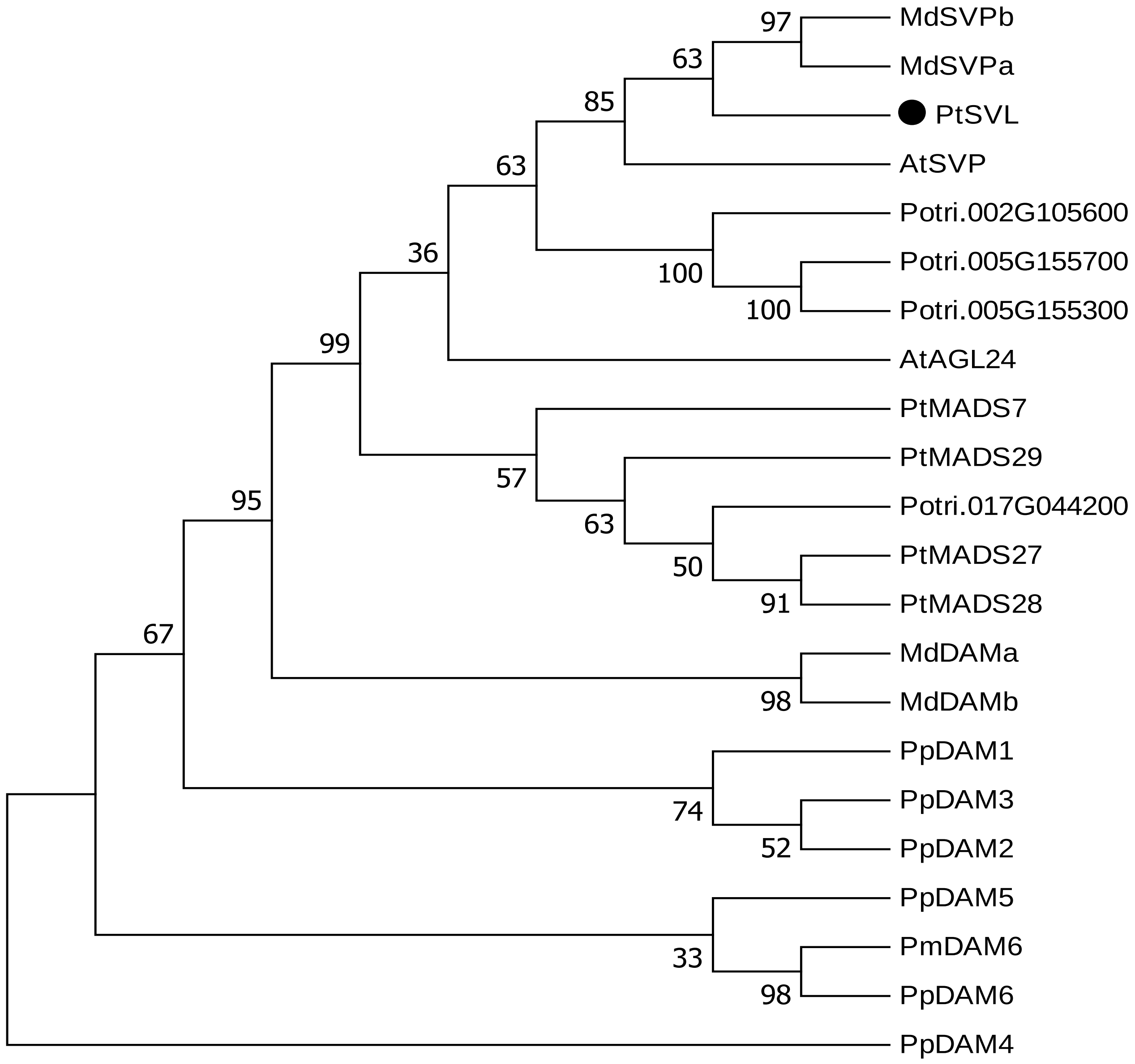


**Supplementary Figure 2. Phylogenetic analysis of Populus *SVL (PtSVL)*.** Phylogenetic tree of Populus SVL (Potri.007G010800) with genes encoding MADS box proteins from Populus including DAM-like proteins (Potri.005G150500 (PtMADS7), Potri.017G044500 (PtMADS27, Potri.007G115200 (PtMADS28) Potri.007G115100 (PtMADS29), and other MADS box proteins similar to SVP (Potri.002G105600, Potri.005G155700, Potri.005G155300, Potri.017G044200), Arabidopsis SVP and AGL24 (AT2G22540, AT4G24540), DAM genes from peach (PpDAM1-6), DAM genes from pear ( PmDAM6) and DAM and SVP-Like genes from Apple ( MdDAM a and b, MdSVPa and b).

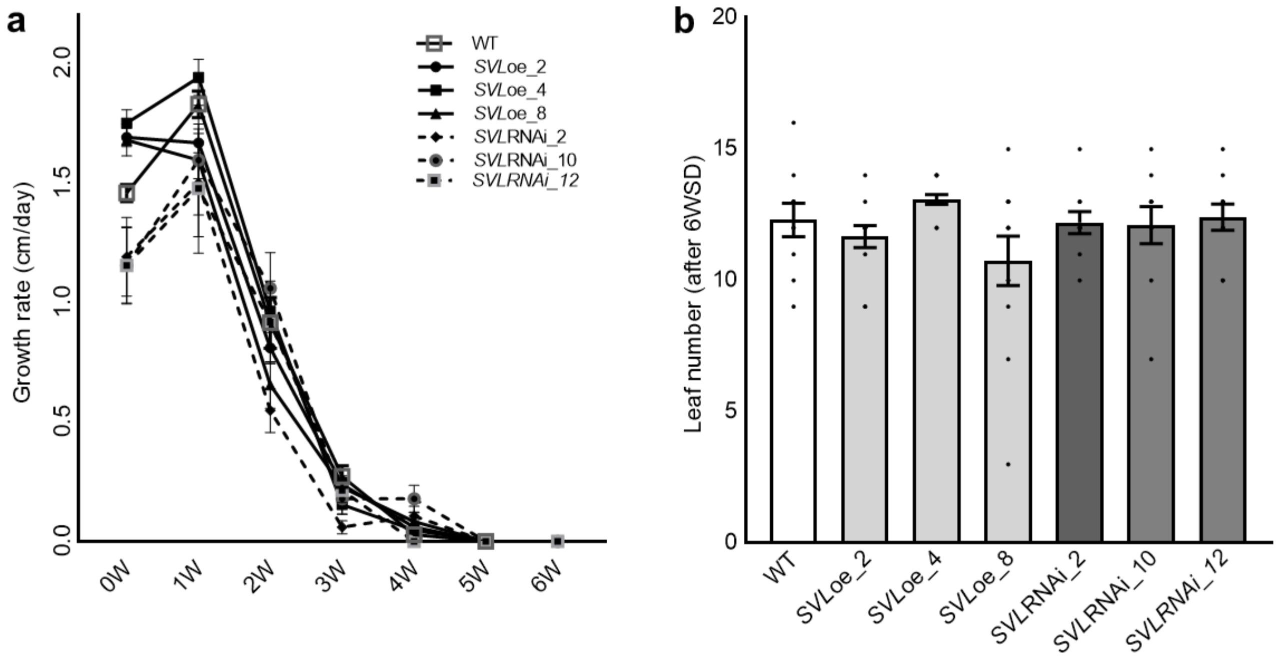


**Supplementary Figure 3. Growth analysis of *SVL*oe and *SVL*RNAi. a** Growth rate and **b** Number of leaves produced by *SVL*oe and *SVL*RNAi under short photoperiod (8h/16h, Light/dark conditions).

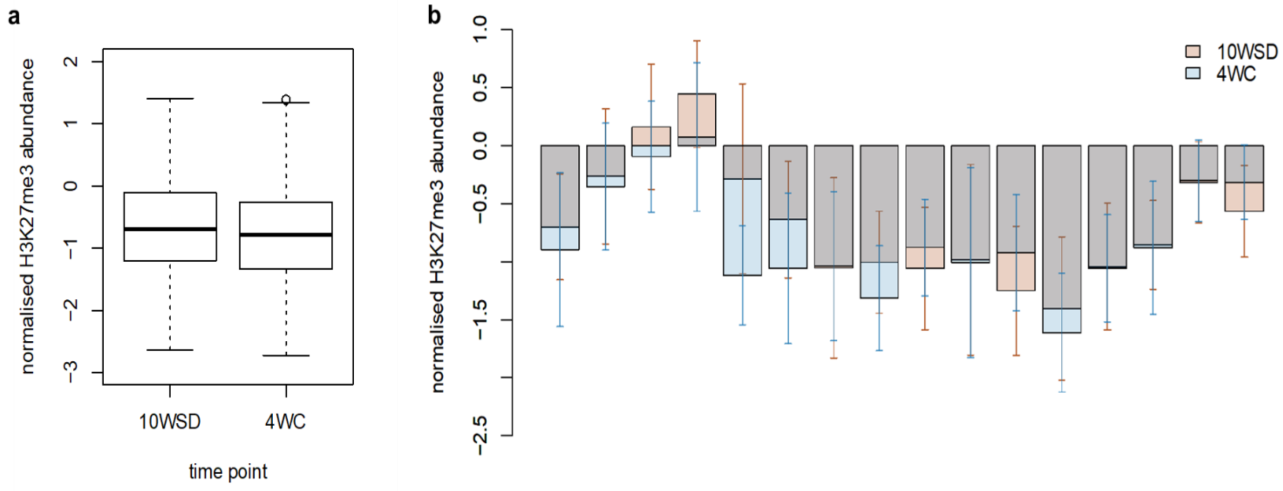


**Supplementary Figure 4. H3K27me3 abundance on *SVL* locus before (10WSD) and after low temperature (4WC). a** Average H3K27me3 abundance of three biological replicates in the *SVL* gene region, including 1 kb downstream and upstream region, was normalised by H3 abundance. Normalised H3K27me3 abundance in the SVL region at the two time points (10WSD and 4WC) is shown. **b** *SVL* gene region, including 1 kb downstream and upstream region, was divided in bins of equal size (546 nt). Gene length is 6737 nt. Abundance of H3K27me3 normalised by H3 is shown for each bin, bars indicate ± standard deviation. Time point 10WSD is shown in brown, time point 4WC is shown in blue and grey colour represents overlapping areas.

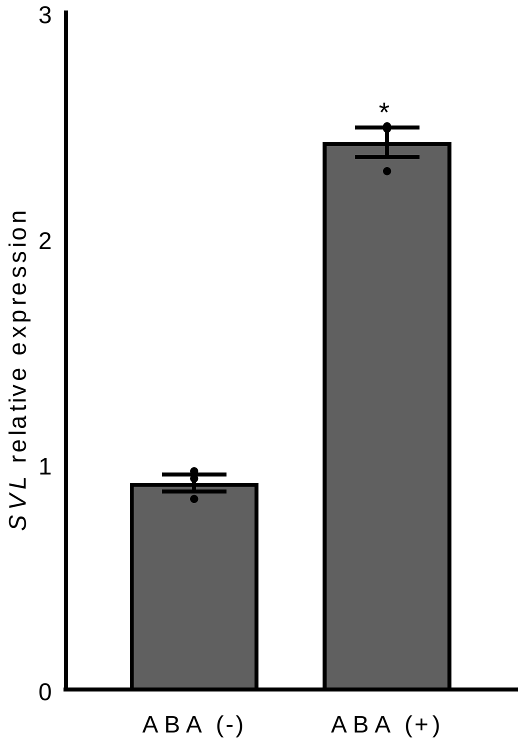


**Supplementary Figure 5.** ***SVL* expression is induced by ABA.** Relative *SVL* transcript levels before and after ABA treatment in wild type (WT) apices. Average expression of *SVL* from three biological replicates ± SEM is plotted relative to the reference gene UBQ and normalized to transcript levels in Non-ABA treated samples set as 1. Asterisks (*) indicate significant difference at, $P<0.005$ from corresponding control calculated using t-test.

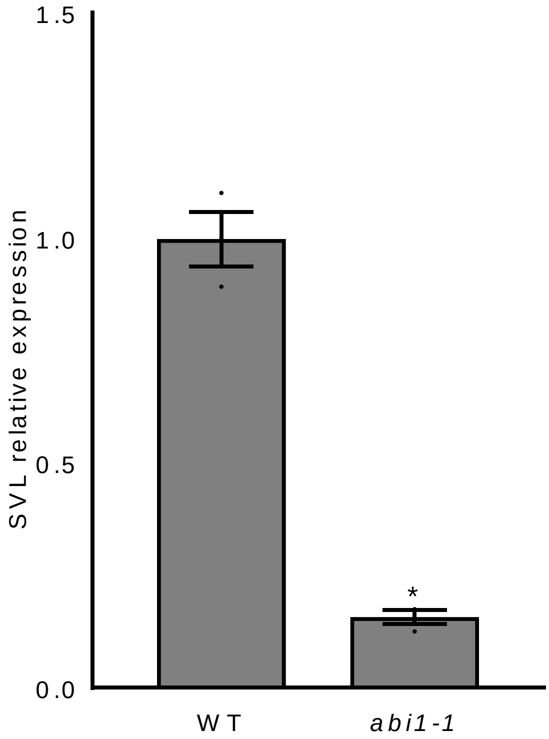


**Supplementary Figure 6. *SVL* expression is altered in ABA insensitive (*abi1-1*) lines.** Relative transcript level of *SVL* in WT and *abi1-1* apices after 10 weeks of short days. Average expression of *SVL* from three biological replicates ± SEM is plotted relative to the reference gene UBQ and with WT expression level set to 1. Asterisks (*) indicate significant difference at, P<0.001 from WT control calculated using t-test.

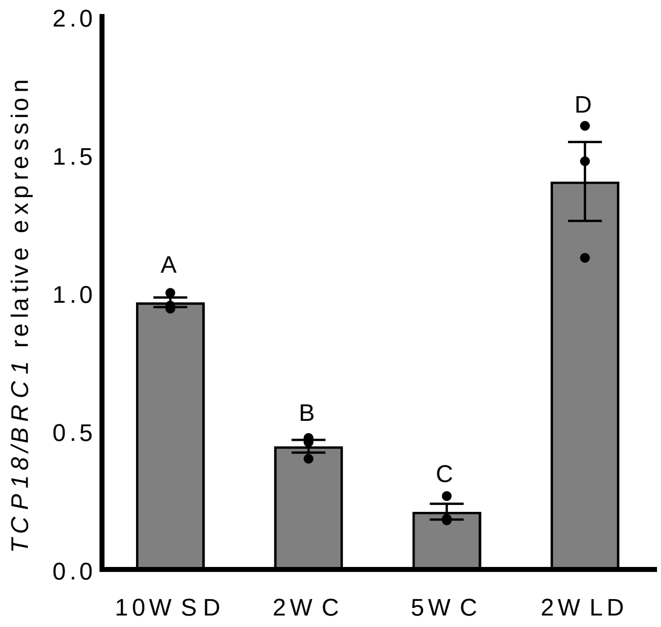


**Supplementary Figure 7. Low temperatures suppress *TCP18/BRC1* expression.** Relative expression of *TCP18/BRC1* after 10 weeks (10W) of short days (SD), followed by 2 and 5 weeks of low temperature (2WC, 5WC at +4$^0$C) and after 2 weeks subsequent exposure to long days and warmer temperatures (2WLD). Average expression of *TCP18/BRC1* from three biological replicates ± SEM is plotted relative to the reference gene UBQ. Different letters (A–D) over the bars indicate significant differences at P<0.001, calculated using Tukey's multiple comparison test.

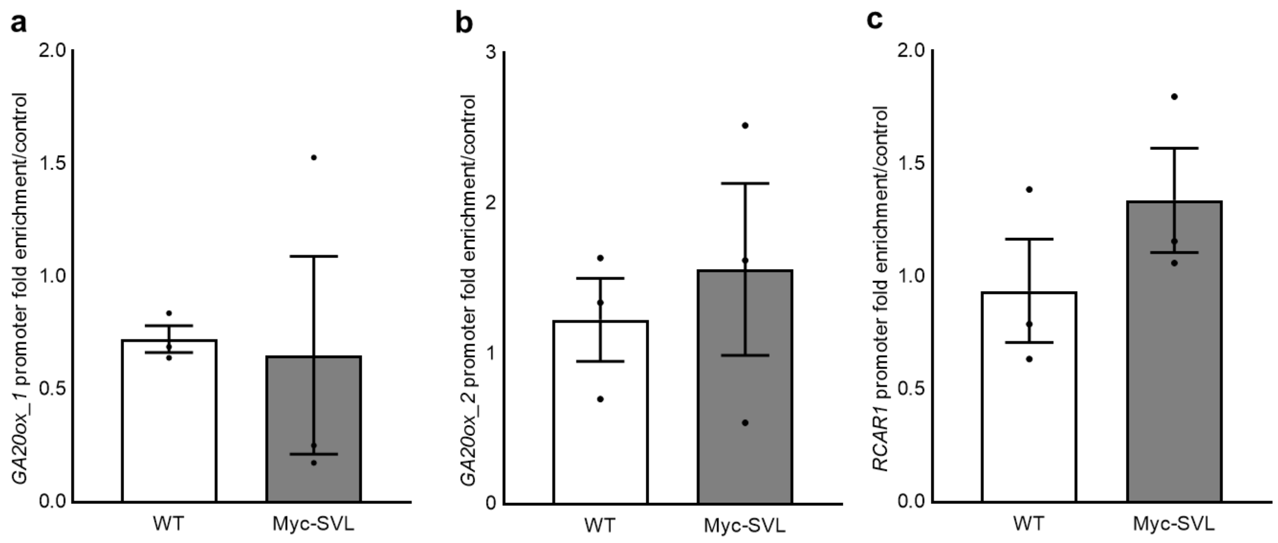


**Supplementary Figure 8.** Enrichment of the DNA fragments containing the putative CArG motif quantified by CHIP-qPCR for **a** *GA20oxidase 1*, **b** *GA20oxidase 2* and **c** *RCAR1* promoter. Presented values were first normalized by their respective input values, then fold enrichments in WT and Myc-*SVL* plants relative to negative controls were calculated. Bars show average values from three independent biological replicates ± SEM.

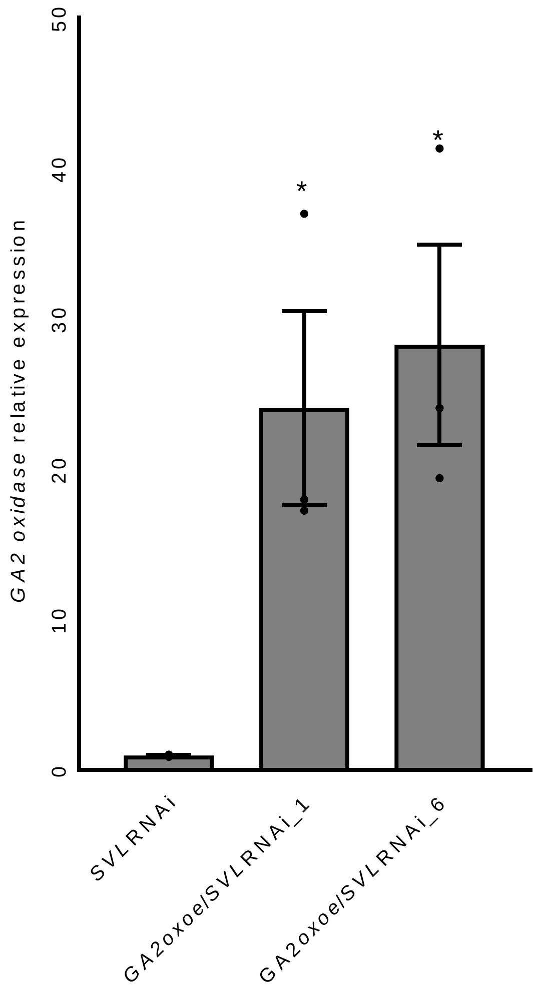


**Supplementary Figure 9. *GA2oxidase* overexpression in *SVL*RNAi plants.** *GA2oxidase* expression in *SVL*RNAi plants and two independent *GA2oxidase* overexpressing lines (1 and 6). Expression of *GA2oxidase* is plotted relative to the reference gene UBQ and normalized to transcript levels in *SVL*RNAi plants. Asterisks (*) indicate significant difference at, P<0.001 from corresponding control calculated using t-test.

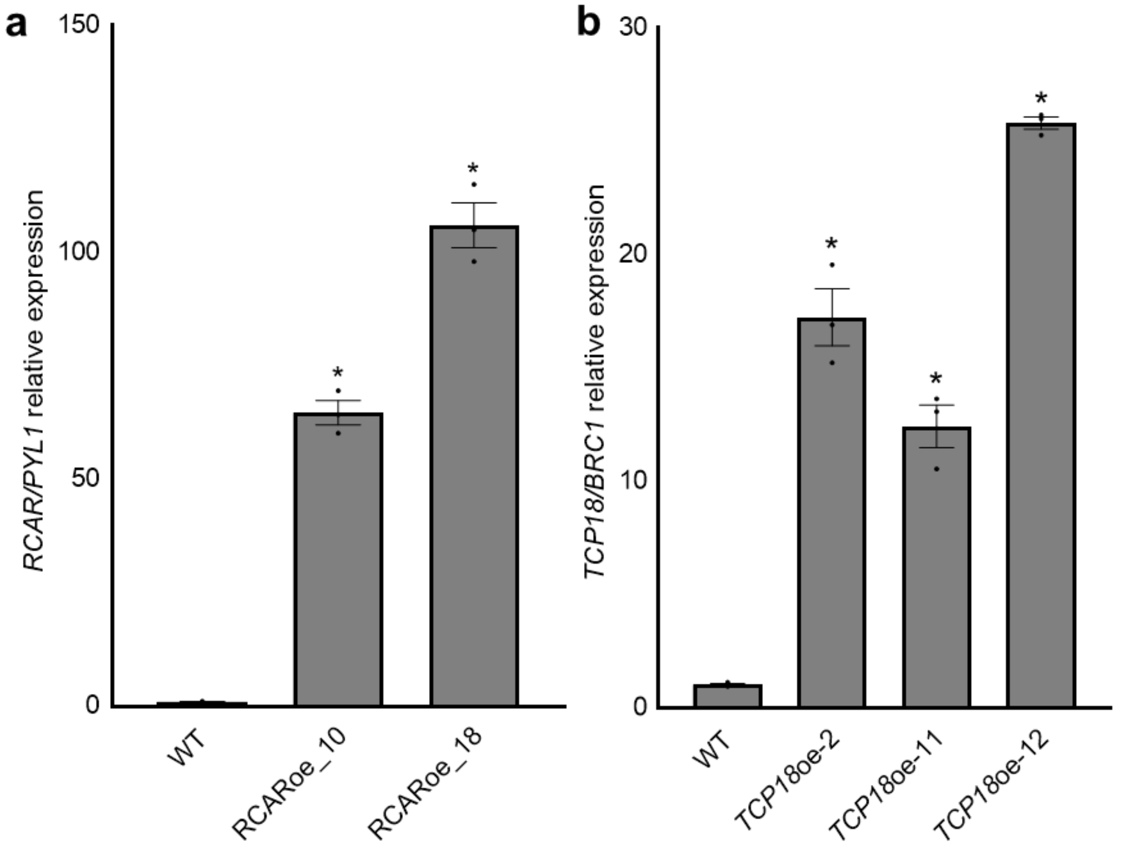


**Supplementary Figure 10.** ***RCAR/PYL1*** **and** ***TCP18/BRC1*** **expression in** ***RCAR*****1oe and** ***TCP18*****oe plants. a** *RCAR/PYL1* expression in WT and two independent *RCAR*oe lines (10 and 18). **b** *TCP18* expression in WT and three independent *TCP18*oe lines (2, 11 and 12). Expression of *RCAR /TCP18* is plotted relative to the reference gene UBQ and normalized to transcript levels in WT controls. Asterisks (*) indicate significant difference at, P<0.001 from corresponding control calculated using t-test.

**Supplementary Table 1. Primers used for cloning and quantitative RT-PCR in the study**

| **Primer Name** | **Sequence** |
|---|---|
| *SVL-Myc Forward (Full length cDNA)* | ATGGGTGAACAAAAGTTGATTTCTGAAGAAGATT TGAACGGTGAACAAAAGCTAATCTCCGAGGAAGA CTTGAACGGTGAACAAAAATTAATCTCAGAAGAAG ACTTGAACGGATCCATGGCAAGAGAGAGGATTC |
| *SVL Reverse (Full length cDNA)* | AAAAAGAATTCTCAGTTTGAAAATGGCAA |
| *SVL Forward (RNAi)* | CACCATGGCAAGAGAGAGGA |
| *SVL Reverse (RNAi)* | GCCAGTGGAGGAGAAGATGATG |
| *RCAR/PYL 1 Forward (Full length cDNA)* | CACCATGAACGGCAGTGATGCCT |
| *RCAR/PYL 1 Reverse (Full length cDNA)* | TCAGAATTGATTGACAGGTTCAAC |
| *TCP18/BRC1 Forward (Full length cDNA)* | CACCATGTTTCCATTAAGCTACAATGCC |
| *TCP18/BRC1 Reverse (Full length cDNA)* | TTAGCACATGCTGCCAATGGTGCAT |
| *GA2-oxidase Forward (Full length cDNA)* | CACCATGGTAGTGGCATCCCCAACTC |
| *GA2-oxidase Reverse (Full length cDNA)* | TCAAGCAACTTGGTCATCTACTTCCAGC |
| *SVL q-PCR Forward* | TGAGAGACTCAAACAGCAAGTGG |
| *SVL q-PCR Reverse* | ACTGCCCTTCCTCGTAACCAAC |
| *FT1 q-PCR Forward* | CAACTGGGGCAAGCTTTGGCCATGAAAC |
| *FT1 q-PCR Reverse* | TTATCGCCTCCTACCACCAGAGCCAC |
| *Ga20-Oxidase1 q-PCR Forward* | TTCCACAACGAGAGCGGTCTTG |
| *Ga20-Oxidase1 q-PCR Reverse* | TTTGAGAGGCAGGGAAGGGAGAG |
| *Ga20-Oxidase2 q-PCR Forward* | GCAGTTCATTTGGCCAGACA |
| *Ga20-Oxidase2 q-PCR Reverse* | CACAGGACTCGCCAATCTTT |
| *RCAR/PYL 1 q-PCR Forward* | AGTGATGCCTACAGTGCAACAGA |
| *RCAR/PYL 1 q-PCR Reverse* | TGCTCACAAATGGCTTGTACCT |
| *RCAR/PYL 2 q-PCR Forward* | AGTGCAACCTGAAGTCACTGG |
| *RCAR/PYL 2 q-PCR Reverse* | CAGGAATCAGCCCCACATAC |
| *NCED3 q-PCR Forward* | TGGAGACCAGAAATTCGGTGGAG |
| *NCED3 q-PCR Reverse* | TGTACCCGTCATCTTCCCTCTCTG |
| *TCP18/BRC1 q-PCR Forward* | TACTGGAGTCATCTGCACAGCAC |
| *TCP18/BRC1 q-PCR Reverse* | AGCACATGCTGCCAATGGTGCAT |
| *FT1 Prom Chip F1* | CGTGGAGCTAACGTGTATGGCG |
| *FT1 Prom Chip R1* | TTCTTTGTCAACTTTCACTCGG |
| *FT1 Prom Chip F2* | TACGAGTTGAAGATGTTACCAC |
| *FT1 Prom Chip R2* | ATGCCCTCCAAATCCATCCTCG |
| *NCED3 Prom Chip F1* | GCCACGTGTAGAGGCAGGTTGCC |
| *NCED3 Prom Chip R1* | ATCCCTCTCCCCACCAATGCTCC |
| *NCED3 Prom Chip F2* | TCAATCCTCAGATCACACAACGC |
| *NCED3 Prom Chip R2* | AAGGTTGAGTGAGTTGTGATCGG |
| *TCP18/BRC1 Chip F1* | ACCATCCACATCAGTAAAACGAC |
| *TCP18/BRC1 Chip R1* | GTTATGATTTGATTAGATATCCC |
| *TCP18/BRC1 Chip F2* | CATTCACAGTCAAAGCAGAGTTGG |
| *TCP18/BRC1 Chip R2* | GGACAAGATTGATCTTATGGTGC |

| | |
|---|---|
| *GA2-oxidase q-PCR Forward* | ATGGTGGTGGCATCTCCAACT |
| *GA2-oxidase q-PCR Reverse* | TCATCGAATGGTTTGGCA |
| *UBQ q-PCR Forward* | GTTGATTTTTGCTGGGAAGCG |
| *UBQ q-PCR Reverse* | GATCTTGGCCTTCACGTTGT |
| *GA20oxidase 1Prom Chip F1* | GAGGTCAATATTTCGAGCAAGC |
| *GA20oxidase 1Prom Chip R1* | TTGTTCATCAAGAAGGCCCACAC |
| *GA20oxidase 1Prom Chip F2* | AATATTTGTGGGTGCATGGAT |
| *GA20oxidase 1Prom Chip R2* | CCATCAGCGAATACCCATTATC |
| *GA20oxidase 2 Prom Chip F1* | TCACAGGGTAATTAGGCCCCTC |
| *GA20oxidase 2Prom Chip R1* | CCCACCTGACATAGGTCCTCTG |
| *GA20oxidase 2Prom Chip F2* | CCACCTCGGTCGGTCCCTCCGC |
| *GA20oxidase 2Prom Chip R2* | CGATGTGCTTAATTACACGGTCC |
| *RCAR1 Prom Chip F1* | GATAAATTGTTGCCAAAGCTTG |
| *RCAR1 Prom Chip R1* | ATTTAAAAGAAGAAAGCACC |
| *RCAR1 Prom Chip F2* | AATATATAAGTGATTGCAC |
| *RCAR1 Prom Chip R2* | TATATTTTCTAACCAATTC |
| *MADS27 q-PCR Forward* | TCGGTTGAGCTTCAGCTTGATAGTGC |
| *MADS27q-PCR Reverse* | CCTTGGACTTGAGAGCATCGACCTCC |
| *MADS28 q-PCR Forward* | AAGGGCATTTGCTCGAACCAGGCCAG |
| *MADS28 q-PCR Reverse* | TAGCCTGCAAGAATTTTGGAGTTGCG |
| *MADS29 q-PCR Forward* | CAATGCTCTCAAGACCAAGGGGGAGC |
| *MADS29 q-PCR Reverse* | TGTCCTGGCGAGGATCAGCTGAGCTC |
| Potri.002G105600 *q-PCR Forward* | AGACAAGTAACCTTCTCCAAGAGGAG |
| Potri.002G105600 *q-PCR Reverse* | AAGCACATCCTTCATACTGGAGCTGG |
| Potri.005G155700 *q-PCR Forward* | GCATTTGAAACAGAAAATGAC |
| Potri.005G155700 *q-PCR Reverse* | TCAGCTTAAGATGGCCAACCC |
